# Routine HIV testing program in the University Infectious Diseases Centre in Lithuania: a four-year analysis

**DOI:** 10.1186/s12879-018-3661-0

**Published:** 2019-01-07

**Authors:** Raimonda Matulionytė, Kęstutis Žagminas, Eglė Balčiūnaitė, Elžbieta Matulytė, Rasutė Paulauskienė, Almina Bajoriūnienė, Arvydas Ambrozaitis

**Affiliations:** 1 Department of Infectious Diseases and Dermatovenerology, Institute of Clinical Medicine, Faculty of Medicine, Vilnius University, Vilnius, Lithuania; 2 Infectious Diseases Centre, Vilnius University Hospital Santaros Klinikos, Vilnius, Lithuania; 3 Institute of Health Sciences, Vilnius University, Vilnius, Lithuania; 4 Centre of Laboratory Medicine, Vilnius University Hospital Santaros Klinikos, Vilnius, Lithuania

**Keywords:** HIV, Routine testing, Targeted testing, Infectious diseases department

## Abstract

**Background:**

HIV transmission remains a major concern in Eastern Europe, and too many people are diagnosed late. Expanded testing strategies and early and appropriate access to care are required. Infectious disease departments might be targets for expanded HIV testing owing to the intense passage of key patient populations that carry indicators of HIV disease. Our objective was to evaluate the feasibility and clinical effectiveness of a fully integrated, opt-out routine, rapid HIV testing program.

**Methods:**

A retrospective four-year study of a screening program was conducted from 2010 through 2014. The program was divided into two periods: from 2010 to 2012 (pilot study) and from 2013 to 2014. The pilot study consisted of routine HIV testing of patients aged 18–55 that were hospitalized in one department. In the second period, all inpatients aged 18–65 were eligible. Targeted testing was conducted in the other inpatient department during the pilot study and the outpatient department during both periods.

**Results:**

During the pilot study, 2203 patients were hospitalized, 1314 (59.6%) were eligible, 954 (72.6%) were tested, and 3 (0.31%) were newly diagnosed HIV-positive. In the second period, 4911 patients were hospitalized, 3727 (75.9%) were eligible, 3303 (88.6%) were tested, and 7 (0.21%) were HIV-positive. In total, 2800 targeted tests were performed, and 4 (0.14%) patients tested positive with newly discovered HIV. All 14 newly diagnosed patients were provided with care. Comparing cumulative groups of routine and targeted testing, the HIV prevalence was 0.23% vs. 0.14% (*p* = 0.40) and was above the reported cost-effectiveness threshold of 0.1% (*p* = 0.012). A lower proportion of advanced disease and a higher proportion of heterosexually transmitted infection were found in the routine testing group.

**Conclusion:**

Routine HIV testing in admissions of infectious diseases is acceptable, feasible, sustainable and clinically effective. Compared to targeted testing, routine testing helped to discover more patients in earlier stages and those with heterosexually transmitted HIV infection.

## Background

Human immunodeficiency virus (HIV) transmission remains a major concern in Europe, in particular the eastern part of the World Health Organization European Region [1]. In Eastern Europe, where the numbers of new HIV infections continue to grow, too many people are diagnosed late, with implications for greater risk for ill health, death and continued HIV transmission [1–5]. The number of new HIV diagnoses has more than doubled over the last decade in the East, and that of AIDS cases has increased by 80%; nearly half of those newly diagnosed with HIV were late presenters [1, 2, 6]. Expanded testing strategies and appropriate and early access to care are required in order to decrease the number of late diagnoses and to support timely linkage to integrated HIV care [3, 7–10].

Lithuania is the largest of the three Baltic States in northeastern Europe. Despite numerous and different historically developed contacts between communities of the three states, the HIV prevalence and rate of spread are fairly different. The estimated HIV prevalence among people aged 15 to 49 in 2015 was 0.2% in Lithuania and 0.6% in Latvia [11], and the rate of HIV diagnosis in Lithuania was 4.8 per 100,000 population in 2014, the lowest of the three Baltic States. For the other two Baltic countries, this rate was highest in the European Union, with 22.1 in Estonia and 17.3 in Latvia, remaining as such in 2015 [6, 7]. Furthermore, Lithuania borders Belarus and the Russian exclave Kaliningrad Oblast, where HIV epidemiological situations are even more threatening. Nevertheless, the rate of HIV diagnosis continued to rise in Lithuania to 7.4 per 100,000 population in 2016. From 1988 to 2016, 2749 HIV cases were diagnosed, 79.3% of whom were men. The major route of transmission was injecting drug use (59.8%), followed by heterosexual contact (21.8%) and sex between men (8.4%) [12]. The percentage of late HIV diseases by CD4 cell count level (< 350 cells per mm^3^ blood) at diagnosis was 66.3% in 2016 and was highest among heterosexuals (69.8%), followed by men who have sex with men (68.0%) and injecting drug users (44.4%) [1]. Eighteen percent of all HIV-infected patients developed AIDS. Despite the general decline in AIDS in the European Union/European Economic Area (EU/EEA) since 2007, an increase was reported in both Lithuania as well as neighboring Latvia [1]. Almost half of all AIDS cases in 2015 and 2016 presented with tuberculosis, and this percentage was the highest in the EU/EEA [1, 6].

“Background testing” in Lithuania consists of physician directed testing in clinical settings; testing on demand in clinical settings and nongovernmental organizations; and routine testing for pregnant women, patients with tuberculosis and sexually transmitted diseases, and patients in substance abuse treatment centers and correctional facilities [13].

Europe is at a crossroads regarding the development of a coherent and comprehensive HIV testing strategy. To facilitate diagnosis and access to HIV-related services, there is a need to move away from patient-initiated HIV testing to additional methods that promote provider-initiated HIV testing [8, 14–16]. Traditional risk assessment methods are thought to be ineffective because clinicians are too busy to perform risk assessment, patients are reluctant to disclose having HIV risk factors even when asked [17], and the potentially discriminatory nature of a testing strategy based on ethnicity or country of origin [8]. Several European countries have advanced new policies to increase uptake, notably through a wider use of routine testing and promotion of opt-out testing [15, 18, 19]. Routinely recommended testing to women in antenatal care has been part of the standard of care in Europe for more than a decade [20, 21]. Guidelines for scaling up HIV testing have been published by some European countries and European Centre for Disease Prevention and Control (ECDC) in high prevalence geographical areas and/or specific populations [14, 15]. A strategy to offer an HIV test to all patients presenting to any healthcare setting with a number of medical conditions, known as HIV indicator conditions (ICs), whether or not they belong to a higher-risk group, has been endorsed across Europe [21–23].

Routine offers of counseling and testing in clinical settings is considered a promising approach to decrease the number of late diagnoses, improve treatment outcomes and prevent further transmission [2, 8]. Along with internationally recognized settings for routine HIV screening, such as sexually transmitted infections, tuberculosis clinics [24, 25] and emergency departments [14, 25–27], infectious diseases departments might also be a desirable target for expanded HIV testing owing to the intense passage of key patient populations sharing the same risk factors with other infectious diseases and carrying clinical indicators for HIV infection. This approach may be of special interest in Eastern Europe, where the health care system is characterized by numerous wards for infectious disease patients, and infectious disease units, as independent centers, are often separated from general emergency departments [28].

The primary goal of our study was to evaluate the feasibility and clinical effectiveness of fully integrated nontargeted opt-out routine rapid HIV testing program. A secondary goal of the study was to determine whether routine testing in an urban teaching infectious disease hospital was associated with identification of more patients with newly diagnosed HIV infection than targeted rapid HIV testing.

## Methods

A retrospective four-year study of a screening program was conducted in Vilnius University Hospital Infectious Diseases Centre from October 1, 2010 to December 31, 2014.

The feasibility was evaluated by HIV testing rates per month. The criteria for clinical effectiveness assessment were HIV disease stage by CD4 count and presence of indicator condition of newly HIV diagnosed patients. Secondary outcomes were the number of patients confirmed to be infected with HIV: 1) newly identified; 2) previously diagnosed but had not disclosed their status before testing procedure. The testing pathway was agreed upon at a joint center meeting attended by hospital administration delegates, physicians and senior representatives from all departments and the laboratory.

### Study center and subjects

The estimated population in Lithuania was 2.9 million people as of 2015, and Vilnius is the capital and the largest city. Vilnius University Hospital Infectious Diseases Centre is an urban teaching hospital that provides care for approximately 2500 inpatients and 5600 outpatients annually. It is the largest of its kind in Vilnius and neighboring districts, serving 27% of the total nation’s population. The hospital consists of an emergency department common for all the wards, first inpatient department (32 beds; generally, care for patients with acute gastrointestinal infections and acute and chronic viral hepatitis), second inpatient department (25 beds; care for patients with all other infections and differential diagnosis issues), intensive care unit, outpatient clinic and laboratory. From 2010 to 2014, the five most common illness groups classified by the International Statistical Classification of Diseases and Related Health Problems 10th Revision – Australian Modification (ICD-10-AM) at discharge were: intestinal infectious diseases (A00-A09), 5348 patients (43.4% of all hospitalized patients); other bacterial diseases (A30-A49), 1933 patients (15.6%); viral hepatitis (B15-B19), 935 patients (7.5%); influenza and pneumonia (J09-J18), 728 patients (5.9%); and viral infections of the central nervous system (A80-A89), 668 patients (5.4%). The center provides the vast majority of primary, consultative and inpatient care to HIV/AIDS in the region, which encompasses approximately one third of the new HIV diagnoses in Lithuania. From 2010 to 2014, 18,272 patients had an outpatient consultation at least once per year. The five most common categories of consultations classified by ICD-10-AM were: viral hepatitis (B15-B19), 5181 patients (28.4% of all consulted patients); intestinal infectious diseases (A00-A09), 2651 patients (14.5%); encounter for screening for infectious and parasitic diseases (Z11), 2201 patients (12.0%); Lyme disease (A69.2), 1750 patients (9.6%); and persons with potential health hazards related to communicable diseases (Z20-Z29), 997 people (5.5%). Additionally, 477 patients (2.6%) were consulted for HIV infection. The hospitals for tuberculosis and sexually transmitted infections are separate from the Infectious Diseases Centre and are under the Pulmonology and Dermatovenerology centers of Vilnius University Hospital, respectively.

The routine HIV testing program during the study period was fully integrated into the hospital operational policy. The program began in October 2010 and was divided into two testing periods differing with regards to operational methods and extent: (1) from October 2010 to December 2012 and (2) from January 2013 to December 2014. The pilot study conducted during the first period consisted of: (1) routine opt-out HIV screening for patients aged 18–55, hospitalized in the second inpatient department; (2) targeted testing for patients hospitalized in the first infectious diseases department; (3) targeted testing for patients consulted in the outpatient clinic and emergency department as outpatients (without successive hospitalization). After 27 months of the pilot study, the importance of routine testing was recognized by hospital administration authorities, and eligibility for routine opt-out testing was expanded. From January 2013, the indications for testing were: (1) routine opt-out HIV screening for all inpatients (hospitalized in the first and second departments) aged 18–65 and (2) targeted testing for patients consulted in the outpatient clinic and the emergency department. Patients determined as having already been infected with HIV or incapable to consent were excluded from the study.

The targeted testing describes physician-directed rapid HIV testing for patients with clinical signs or symptoms similar to those of HIV infection. Additionally, physicians occasionally targeted patients considered to be at increased risk on the basis of actual or antecedent behavioral, sexual or social characteristics. Patients consulted for confirmation of HIV infection tested positive in another setting or for already confirmed HIV infection, and contacts of HIV-infected patients were not considered as subjects of targeted testing and were not included in the study.

### Testing procedures

Prior to admission to specialized wards, patients were assessed in the emergency department where routine HIV testing was proposed for the eligible patients with intended hospitalization. Emergency department physicians were instructed and trained to inform the eligible patients that they would be tested for HIV in line with other medical testing, and assent was inferred unless the patient declined. The patients were offered the opportunity to ask questions or to decline testing. A blood sample for testing was taken in one of the two departments. After hospitalization, the physician of the ward revised all the prescriptions for testing and added the needed tests, including a test for HIV by default, according to the approved procedure. Targeted testing was stated to be assigned by either an emergency or ward physician by their own decision. The emergency department and other center staff (medical doctors and residents) were also trained to answer all arising questions concerning testing, HIV infection and the meaning of positive or negative test results. Consent for HIV testing was incorporated into the patient’s general informed consent for medical care on the same basis as other screening or diagnostic tests.

The usual pro-forma for HIV testing was adapted to allow staff to document the test offer. Patients with negative results were informed by a physician of the department where the patient was hospitalized without systematic provision of posttest counseling. Positive results were given in person and in confidence by senior physicians only after receiving a written form with a confirmed result from the laboratory. An HIV specialist follow-up was arranged, ensuring a link to HIV care in the same institution.

Data for analysis about all the prescribed tests and their results were captured in the laboratory electronic database. A retrospective four-year analysis was conducted. Testing rates and outcomes of those testing HIV seropositive were determined by reviewing hospital databases and medical records.

### The diagnosis of HIV: Testing and confirmation

Rapid Immunochromatographic Card Test FIRST RESPONSE® HIV 1–2.O CARD TEST (Premier Medical Corporation Ltd., Daman, 396,215.INDIA) was used with a serum sample taken together with samples for other laboratory analyses. The test detects antibodies to both HIV-1 and HIV-2 and provides the result in 5–15 min with few manipulations. If reactive, the patient had blood drawn and sent to the National Public Health Surveillance Laboratory for confirmatory testing by Western blot or indirect immunofluorescence assay (IFA). Patients presenting with nonspecific symptoms related to acute retroviral illness, including fever, rash, pharyngitis or lymphadenopathy, were tested with a 4th generation antibody/antigen enzyme-linked immunosorbent assay irrespective of the rapid antibody test response, and a sample was sent to the National Public Health Surveillance Laboratory for confirmation with p24 antigen neutralization reaction and (or) nucleic acid amplification testing.

### Statistical considerations

Seroprevalence with Exact 95% Fisher’s confidence interval was reported. Categorical data were analyzed using the Pearson χ2 or Fisher’s exact test, and Wilcoxon rank sum test was used for continuous variables. Data for categorical variables were reported as the percentages and for continuous variables as the medians (range). Statistical analyses were conducted using Stata (StataCorp. 2011. Stata Statistical Software: Release 12. College Station, TX: StataCorp LP.).

## Results

During the pilot study period (27 months), there were 5827 hospitalized patients; 2203 patients were hospitalized in the second department, 1314 (59.6%) were eligible (age 18 to 55) for routine HIV testing and 954 (72.6%) were offered a rapid test. Among the tested patients, all were white, 540 (57%) were male and the median age was 32. Four patients (0.42, 95% CI 0.11–1.07) tested HIV-positive, of whom 3 (0.31, 95% CI 0.06–0.92), one of them male, were newly diagnosed.

During the 9 months prior to the study, 774 patients were hospitalized in this department, 44 tests were performed for 5.7% of patients and no tests (0, 95% CI 0.00–8.04) were HIV-positive. This resulted in a 7.5-times increase in the HIV testing rate per month during the pilot study (*p* < 0.0001).

**Fig. 1:**
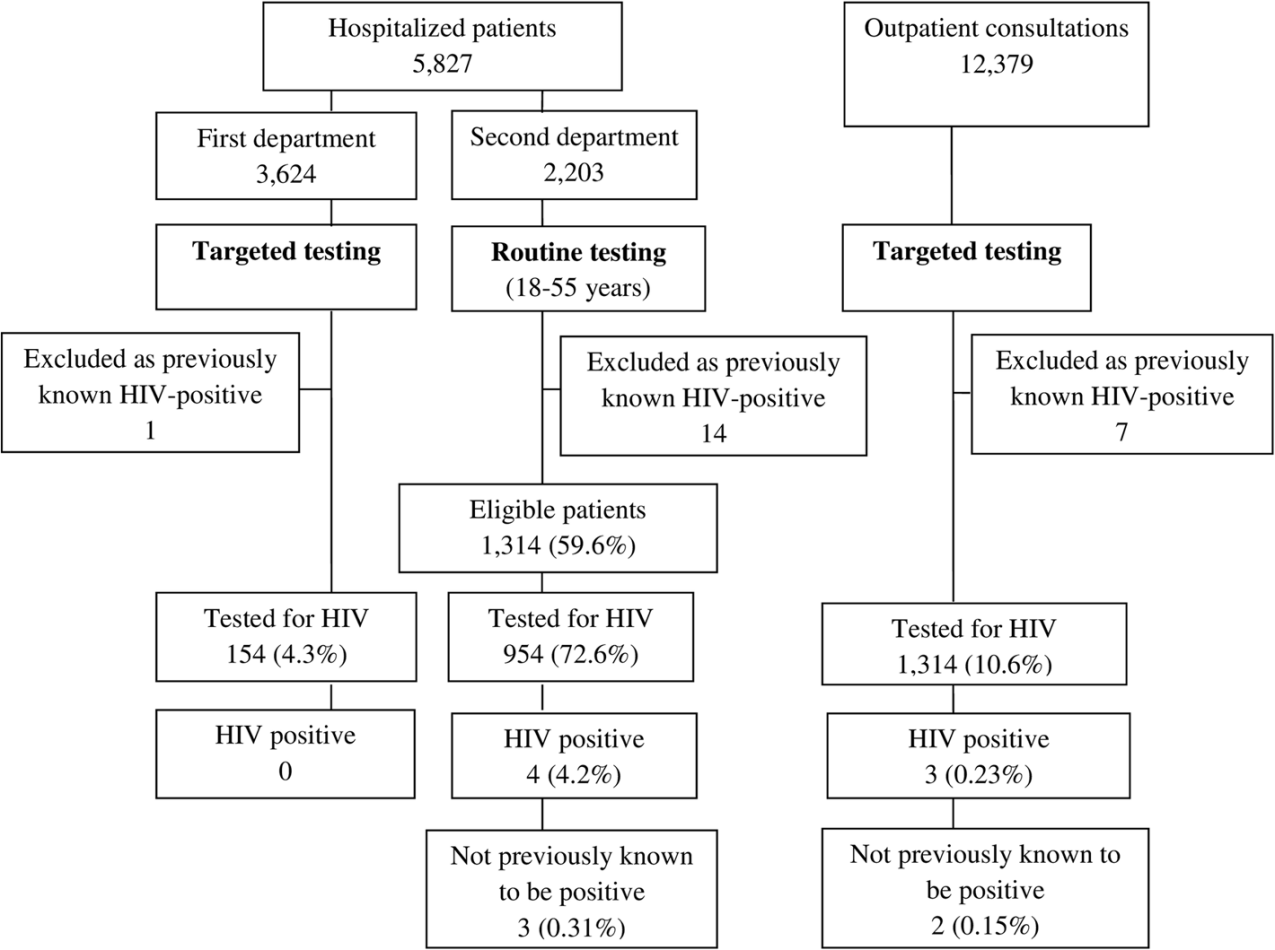
Flow chart showing the numbers of patients and outcomes in the first (pilot) study period (October 2010 to December 2012)

Targeted testing was conducted in the first department where 3624 patients were hospitalized over 27 months; 154 patients (4.3%) were tested for HIV, all were white, 90 (58%) were male and the median age was 36. None of the tested patients (0, 95% CI 0.00–2.37) was HIV-positive (Fig. 1).

**Fig. 2:**
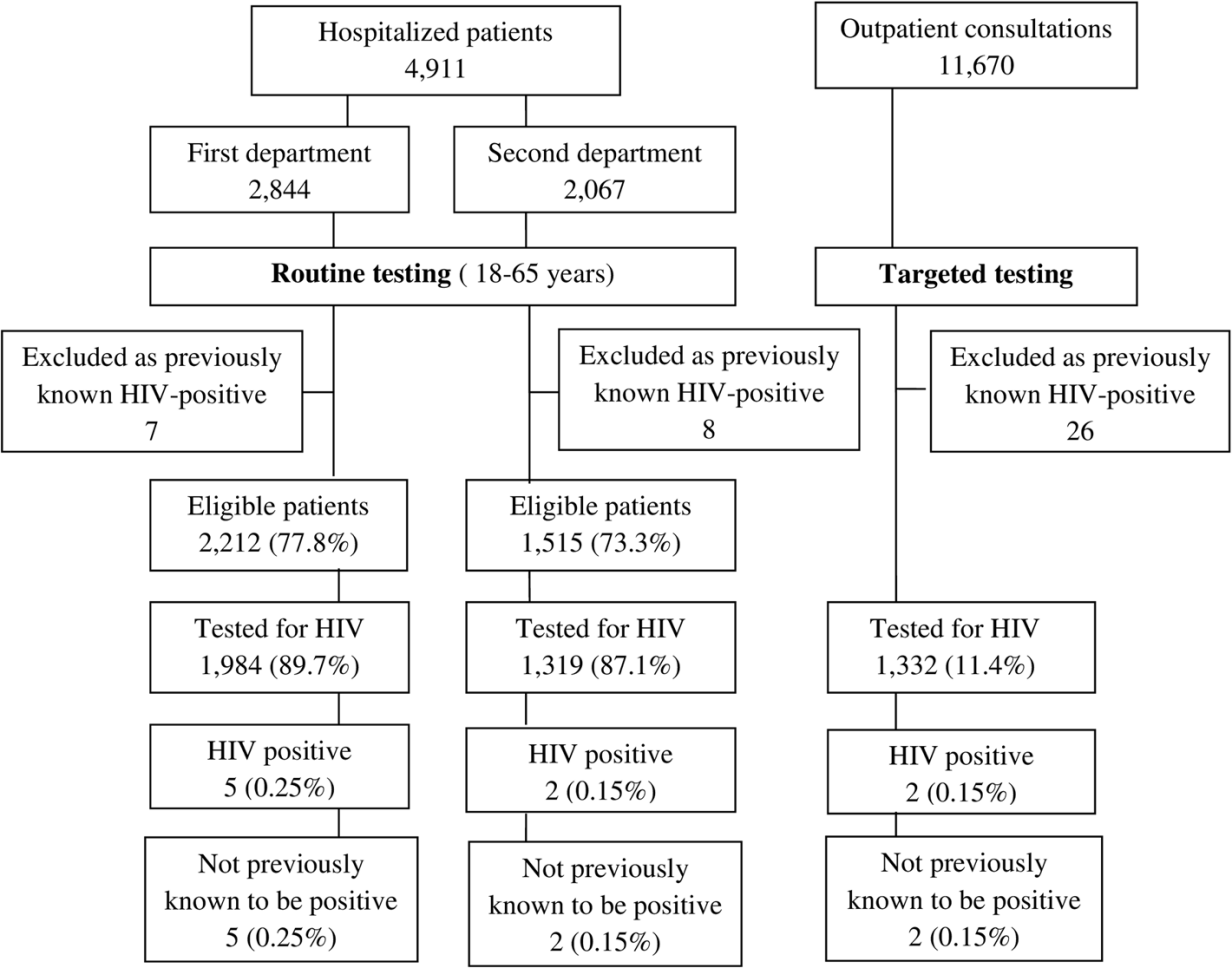
Flow chart showing the numbers of patients and outcomes in the second study period (January 2013 to December 2014

In the second period (24 months), routine testing was conducted in both departments. In total, 4911 patients were hospitalized, 3727 were eligible (aged 18 to 65) for routine testing and 3303 (88.6%) were tested. Among the tested patients, all were white, 1553 (47%) were male and the median age was 33. Seven patients (0.21, 95% CI 0.09–0.42), 4 of them male, had a positive confirmed result, all with a new HIV diagnosis (Fig. 2).

There was a 16.6-times increase in HIV testing on average per month in the first department in the second study period compared to the first period when targeted testing was conducted (*p* < 0.001). The test rate in the second department increased from 72.6% in the pilot period to 87.1% in the second period (*p* < 0,001) but was lower compared to that in the first department (89.7%, *p* = 0.013) during the second period.

Targeted testing was conducted in the outpatient clinic during both study periods. Overall, 24,049 patients were consulted in the clinic; 2646 patients (11.0%) were tested for HIV, all were white, 1540 (55%) were male and the median age was 37. Five patients (0.19, 95% CI 0.06–0.44), all male, tested HIV-positive, and four of them were newly diagnosed (0.15, 95% CI 0.04–0.39) (Figs 1 and 2). During the first study period, targeted testing in the outpatient department was conducted in parallel with targeted testing in the first department, but the percentage of patients tested was significantly higher in the outpatient department (10.6% vs. 4.3%, *p* < 0.001) (Fig. 1).

Table 1 summarizes the main characteristics of the study population stratified in the cumulative groups of routine and targeted testing during both study periods. Women made up a significantly higher percentage among the routinely tested patients (51% vs. 45%, *p* < 0.001), and both groups differed in age distribution. HIV prevalence was 0.23% (95% CI 0.11–0.43) in the routinely tested group and 0.14% (95% CI 0.04–0.37) in the targeted testing group (*p* = 0.40).

**Table 1:** Comparison of patient characteristics and testing results in the cumulative routine and targeted testing groups during both study periods from October 2010 to December 2014

	Routine testing (*n* = 4257)	Targeted testing (*n* = 2800)	p
Sex, n (%)			
Female	2164 (51%)	1260 (45%)	< 0.001
Male	2093 (49%)	1540 (55%)	
Age groups (years), n (%)			
18–25	1159 (27%)	437 (16%)	< 0.001
26–35	1288 (30%)	855 (31%)	
36–45	725 (17%)	596 (21%)	
46–55	699 (16%)	509 (18%)	
56–65	386 (9%)	299 (11%)	
65+		104 (4%)	
Newly identified HIV, n (%)	10 (0.23%)	4 (0.14%)	0.40

Table 2 shows the causes of hospitalization or consultation of patients and who were newly discovered with HIV infection according to the tested group. In the pilot study, routinely tested patients with newly diagnosed HIV infection had *Herpes zoster*, mononucleosis-like illness and erysipelas as causes of hospitalization. In the second period, the causes were *Salmonella enteritidis* sepsis, mononucleosis-like illness, unexplained weight loss, acute enteritis for 2 patients (due to *S. enteritidis* and *Campylobacter* sp.), and chronic hepatitis C virus infection for 2 patients. In the outpatient department, patients with newly discovered HIV were consulted for mononucleosis-like illness, unexplained weight loss, chronic *H. simplex* infection and chronic hepatitis C virus infection.

**Table 2:** Causes of hospitalization or consultation of patients with newly diagnosed HIV infection (*n* = 14)

Condition or disease	Number		
	Routine testing (*n* = 10)	Targeted testing (*n* = 4)	Total (*n* = 14)
AIDS defining conditions	1	1	2
- *Salmonella enteritidis* sepsis	1		
- *Herpes simplex* ulcer		1	
Conditions associated with an undiagnosed HIV prevalence of > 0.1%	6	3	9
- *Herpes zoster*	1		
- Unexplained weigh loss (wasting syndrome)	1	1	
- Mononucleosis like illness (primary HIV infection)	2	1	
- Chronic HCV infection	2	1	
Conditions not classified as indicator for HIV	3	–	3
- Acute enteritis without bacteriemia due to:			
*S. enteritidis*	1		
*Campylobacter sp*.	1		
- Erysipelas	1		

Comparing the characteristics of the HIV-positive patients in the cumulative routine and targeted testing groups, a lower proportion of advanced disease (CD4 < 200/mm^3^) was found in the routinely tested group (2/10 vs. 4/4, *p* = 0.015) as well as a higher proportion of heterosexually transmitted infections (8/10 vs. 1/4, *p* = 0.041). No significant differences were found in age distribution, although all 4 patients in the targeted testing group were < 45 years of age compared to 6/10 in the routine testing group. All the patients diagnosed with HIV in both groups were linked to care in our center. Nine out of 10 patients from the routine testing group and all the 4 patients from the targeted testing group were not lost to follow-up after 6 months. Three contact persons of newly diagnosed patients (one per patient) in the routine testing group and one in the targeted testing group were identified as HIV-positive (Table 3).

**Table 3:** Comparison of newly identified HIV infected patient characteristics in routine testing and targeted testing groups

	Routine testing (*n* = 10)	Targeted testing (*n* = 4)	p
Age groups (years)			
18–25	1	0	0.63
26–35	4	2	
36–45	1	2	
46–55	2	0	
56–65	2	0	
Gender			
Male, n (%)	5 (50%)	4 (100%)	0.22
Female, n (%)	5 (50%)	0	
Transmission group, n (%)			
Intravenous drug usage	2 (20%)	1 (25%)	0.041
Heterosexual	8 (80%)	1 (25%)	
Men who have sex with men	0	2 (50%)	
Disease stage, n (%)			
CD4 count < 200/mm^3^	2 (20%)	4 (100%)	0.031
CD4 count 200–350/mm^3^	3 (30%)	0	
CD4 count > 350/mm^3^	5 (50%)	0	
CD4 cell count:			
Min	65	58	0.088
Max	771	137	
Median	413.5	95	
Interquartile range	360	71.5	
Patients for whom HIV positive contacts were detected, n (%)	3 (30%)	1 (25%)	0.999

## Discussion

Our experience shows that routine HIV testing in infectious disease admissions is acceptable, feasible, sustainable and clinically effective. The results show good acceptability of routine testing with rapid tests. In addition to high testing rates per month, good feasibility has also been proven by wide acceptance by the hospital administration and medical staff. There was also growth in the testing rate during routine testing itself, which showed an increased testing rate in the second period in the second department. The general prevalence of previously undiagnosed HIV infection found by routine testing was 0.23%. Although our study was not focused on the evaluation of cost-effectiveness in the frame of local resources, the strategy might be cost-effective, with a prevalence above the reported cost-effectiveness threshold of 0.1% [16, 18]. The implementation of routine HIV testing in one department dramatically increased HIV testing rates during the pilot study, and 0.3% of the routinely tested 18–55-year-old patients were HIV-positive compared to none during parallel targeted testing in the other department. A lower targeted testing rate (4.3%) was seen in this inpatient department compared to outpatient consultations (10.6%), where the same testing strategy was used in parallel during the first period. Some possible explanations are as follows: 1) different patient populations in inpatient and outpatient sectors: one of the best defined HIV indicator diseases, viral hepatitis, led to nearly a third of outpatient consultations but only of 7.5% of hospitalizations, and some hospitalized patients had already been tested as outpatients before hospitalization; 2) different staff members’ experiences and convictions of the utility of testing for HIV, knowing that the personnel of this inpatient department provided care mostly to patients with acute gastrointestinal infection and had less experience in testing and recognizing HIV specific situations. This is different from physicians in the outpatient department who provide care for patients with different acute and chronic pathology frequently overlapping with well-defined ICs, as well as for patients already diagnosed with HIV; 3) implicit selection of patients for testing by less experienced staff due to their longstanding habit of prioritizing testing based on risk factors, knowing that the majority of diagnosed HIV cases in Lithuania were transmitted among injecting drug users.

The high test acceptability rate, the results and the motivation of the staff during the pilot study encouraged us to reinforce routine testing in the second period, with involvement of all patients requiring hospitalization in infectious disease wards and enlargement of age limits to 18–65. This led to a dramatic 16.6-times increase in the test-offering rate in the first department, which resulted in an HIV prevalence of 0.25% in the second period. Driven by similar reasons, the staff of the second department, where routine testing had already been started during the first period, strengthened their attempts to offer more tests, which significantly expanded the testing rate from 72.6 to 87.1%.

Comparing cumulative groups of patients tested routinely (*n* = 4257) with those targeted due to clinical or epidemiological suspicion (*n* = 2800), a significantly higher women’s percentage was found among the routinely tested patients, and both groups were different by age distribution. The rate of newly identified HIV-positive patients was comparable (0.23% vs. 0.14%, *p* = 0.40), although a trend toward higher HIV prevalence among routinely tested patients was observed. Routine testing discovered more cases of HIV infection transmitted heterosexually and with higher CD4 counts at presentation compared to targeted testing, and contributed to the detection of HIV infections in older and female patients. None of female patients was HIV-positive in the targeted testing group during either study period. This correlated with previous findings that patients not belonging to the men who have sex with men HIV-exposure group, females or those of older age are implicitly less likely to be offered the test, which inevitably leads to later presentation for HIV care [2, 29–32]. Epidemiological data in Europe, especially in the East, have shown that a significant number of people are infected through heterosexual contact, a high percentage of whom of late presenters, enhancing the importance of testing programs contributing to diagnosing patients belonging to those categories [2, 6]; our program conformed well to these initiatives. Furthermore, detecting contacts of newly diagnosed HIV-infected patients additionally increased the yield of the strategy. All patients were linked to care, and all but one remained in care 6 months later. The facilitated linkage process reflects a privilege to be diagnosed with HIV directly in an infectious disease setting.

The cost-effectiveness and practicability of universal “opt-out” testing have been the subject of continuous debate [29, 33–35]. Nevertheless, during the last decade, emergency physicians have increasingly recognized the importance of emergency departments in HIV diagnosis and prevention [26, 27, 36–38]. Although routine screening feasibility in clinical venues and its cost-effectiveness have been demonstrated from a societal perspective [16], debates over cost-effectiveness and the impact on public health are ongoing, and we must work to understand which screening strategies are most effective to identify patients with HIV infection [30, 38].

With regards to a risk assessment-based strategy, a low perception of individual risk and fear is an important barrier to testing [8, 10, 25]. Consequently, it often depends on subjective assessment of risk by clinicians [8, 17]. The sensitivity aspect of risk assessment has been of special consideration in Eastern Europe, where disclosure of homosexuality remains highly stigmatized. Only 2% of HIV infections reported to the ECDC in 2014, and 4% - in 2015 [6], consisted of transmission through sex between men in Eastern Europe [7], and almost all (97%) the individuals from East Central Europe enrolled in HIV Indicator Diseases across Europe study 1 (HIDES 1) reported their sexual orientation as heterosexual compared to 60% in other regions [22].

The IC-based strategy proven to be clinically effective helps to identify earlier-stage HIV infection and is acceptable to patients and healthcare practitioners [39]. However, our results indicate that nearly a third of patients discovered HIV-positive by routine testing had no evidence of broadly accepted HIV indicators of disease or conditions at presentation [21]. Diarrhea as a cause of hospitalization of two patients could not have been classified to be chronic or unexplained and could have been missed if patients with only indicator conditions had been tested. Discussing existing studies in Europe, HIV ICs are not always suitably exploited as triggers for early HIV testing and are inadequately applied. In a number of cases, persons diagnosed late with HIV had been in contact with the healthcare system [10, 29, 33, 39]. The HIDES 2 Study showed that test offer rates in well-established HIV ICs remained low across Europe, reflecting missed opportunities for earlier HIV diagnosis and care [3]. A recent evaluation of HIV testing recommendations in specialty guidelines in the United Kingdom demonstrated that the majority of specialty guidelines for ICs do not yet recommend testing for HIV, and clinicians managing ICs may be unaware of national recommendations produced by HIV societies or the prevalence of undiagnosed HIV infection among patients with ICs [40].

Infectious disease departments can be suitable settings for routine HIV testing owing to the intense passage of key patient populations sharing the same risk factors as other infectious diseases and carrying indicator conditions and indicator-like conditions regardless of their interpretation until definitive diagnosis. Our program was highly acceptable to both patients and staff, dramatically increased testing rates, improved medical care provider attitudes toward HIV and raised awareness about HIV testing and its clinical indications. Patients diagnosed with HIV were not all from high risk groups, which suggests that the scope of universal testing should be extended. Routine testing in infectious disease admissions is especially favorable for newly HIV diagnosed patients’ linkage to all phases of care, re-engagement of previously known and disengaged patients, identification of infected contact persons, and retention in care processes. The high acceptability rate in our study demonstrated that individuals are more likely to be tested for HIV when they can communicate on the subject without restraint and perceive more benefits from testing [25, 41]. The shift away from HIV exceptionalism and social stigma toward normalization of testing suggests that HIV should be treated more similarly to other infectious diseases, requiring early diagnosis to ensure timely treatment and prevention. The added value of the program effectuated in a university hospital is that it improved our ability to recognize early clinical indicators and identify risk factors and served for medical training at all academic levels.

This study has several limitations. There was heterogeneity between populations in the cumulative groups of targeted and routine testing. The study design of opt-out testing introduces the risk of selection and information bias for study outcomes. First, it was not possible to ascertain either an exact test refusal rate or reasons for refusal as it was not documented. The missing HIV tests can be biased by physician factors related to not assigning a rapid HIV test for a patient. Second, inpatient and outpatient populations were fairly different for prevalence results, and our targeting criteria were not used as an instrument to systematically assess risk and were principally based on a physician’s own decision to provide a rapid test for a patient. Therefore, our study reflects the actual extent of targeted testing based on a physician’s decision in routine clinical care in infectious disease admissions. Finally, we used third-generation HIV rapid diagnostic tests, the backbone of testing in most low- and middle-income countries [42, 43], missing the earliest preantibody phase. Only patients presenting with nonspecific symptoms related to acute retroviral illness were tested with the 4th generation antibody/antigen test, regardless of the rapid antibody test response. This could have led to some acute HIV infection diagnoses being missed due to confusion with other differential conditions.

## Conclusions

Routine HIV testing in infectious diseases admissions is acceptable, feasible, sustainable and clinically effective. Compared to targeted testing, routine testing helped to discover more patients in earlier stages and those with heterosexually transmitted HIV infection who might have been missed with targeted testing. Expanded testing in infectious diseases settings is favorable for linkage to care, under- and postgraduate educational process and stigma reduction and is of special worth to intensify IC-based HIV testing during the transitional period until IC-guided testing methodology is applied in all health care sectors. More prospective studies are needed to support systematic application of this strategy. Nevertheless, we believe that routine testing in infectious diseases wards can improve the early detection of persons less likely to be diagnosed by targeted strategies and change the attitude of professionals toward HIV testing normalization.

## Abbreviations

ECDC: European Centre for Disease Prevention and Control
EU/EEC: European Union/European Economic Area
HIV: Human immunodeficiency virus
ICD-10-AM: International Statistical Classification of Diseases and Related Health Problems 10th Revision – Australian Modification
ICs: Indicator conditions
IFA: Immunofluorescence assay
